# circPTN sponges miR-145-5p/miR-330-5p to promote proliferation and stemness in glioma

**DOI:** 10.1186/s13046-019-1376-8

**Published:** 2019-09-11

**Authors:** Jiansheng Chen, Taoliang Chen, Yubo Zhu, Yan Li, Yuxuan Zhang, Yun Wang, Xiao Li, Xiaomi Xie, Jihui Wang, Min Huang, Xinlin Sun, Yiquan Ke

**Affiliations:** 1 The National Key Clinical Specialty; Department of Neurosurgery, Zhujiang Hospital, Southern Medical University, Industrial Road No.253, Guangzhou, 510282 Guangdong China; 2 Department of Neurosurgery, Huizhou Municipal Central Hospital, Huizhou Shi, China

**Keywords:** circPTN, Glioma, miR-145-5p, miR-330-5p, GSCs

## Abstract

**Background:**

Growing evidences indicate that circular RNAs (circRNAs) play an important role in the regulation of biological behavior of tumor. We aim to explore the role of circRNA in glioma and elucidate how circRNA acts.

**Methods:**

Real-time PCR was used to examine the expression of circPTN in glioma tissues and normal brain tissues (NBT). Assays of dual- luciferase reporter system, biotin label RNA pull-down and FISH were used to determine that circPTN could sponge miR-145-5p and miR-330-5p. Tumor sphere formation assay was performed to determine self- renewal of glioma stem cell (GSCs). Cell counting Kit-8 (CCK8), EdU assay and flow cytometry were used to investigate proliferation and cell cycle. Intracranial xenograft was established to determine how circPTN impacts in vivo. Tumor sphere formation assay was performed to determine self- renewal of glioma stem cell (GSCs).

**Results:**

We demonstrated circPTN was significantly higher expression in glioma tissues and glioma cell lines, compared with NBT and HEB (human astrocyte). In gain- and loss-of-function experiments, circPTN significantly promoted glioma growth in vitro and in vivo. Furthermore, we performed dual-luciferase reporter assays and RNA pull-down assays to verify that circPTN acts through sponging miR-145-5p and miR-330-5p. Increasing expression of circPTN rescued the inhibition of proliferation and downregulation of SOX9/ITGA5 in glioma cells by miR-145-5p/miR-330-5p. In addition, we found that circPTN promoted self-renewal and increased the expression of stemness markers (Nestin, CD133, SOX9, and SOX2) via sponging miR-145-5p. Moreover, this regulation was disappeared when circPTN binding sites in miR-145-5p were mutated.

**Conclusions:**

Our results suggest that circPTN is an oncogenic factor that acts by sponging miR-145-5p/miR-330-5p in glioma.

**Electronic supplementary material:**

The online version of this article (10.1186/s13046-019-1376-8) contains supplementary material, which is available to authorized users.

## Backgroud

Glioma, which is one of the most prevalent brain tumors, is a refractory disease even after conventional therapy [1]. Patients who are diagnosed with glioblastoma (GBM), the most malignant glioma, have a median survival time of approximately 1 year [2]. Improving the early diagnosis of GBM and developing advanced treatments based on a greater understanding of the biogenesis of GBM are important research goals. Recently, studies have unveiled the role of circular RNA (circRNA) in cancer pathogenesis. CircRNAs were first discovered in eukaryotic cells in 1979 (Hsu MT et al. [3]), but these RNA did not raise much attention at that time. With the development of deep-sequencing technology, Memczak et al. showed that circRNAs were a new class of RNA in animals, and demonstrated the back-spliced formation of circRNAs and described ciR-7, which is one of the most abundant circRNAs, as essential to the development of the mesencephalon by sponging miR-7 (Memczak et al. and Hansen TB et al. [4, 5]). Unlike linear RNA, circRNA exists as a closed loop without a 3′ polyadenylate tail or 5′ cap and is therefore more stable and resistant to RNase R [6]. In addition, circRNAs are tissue- specific [7–10]. Although the mechanism underlying the action of circRNAs is unknown, certain circRNAs have been reported to function via microRNA (miRNA)-sponging [11–19], binding to RNA-binding protein (RBP) [20–22], or by interacting with RNA-pol II to regulate transcription [23] and translation of peptides and proteins [24–27].

Song et al. sequenced circRNAs in 27 glioma tissues (20 GBMs and 7 oligodendrogliomas) and in 19 samples of normal brain tissue (NBT) [28]. Based on their study, we screened several circRNAs that were upregulated in glioma tissues compared with NBT. Considering the expression profiles in glioma cell lines compared with normal astrocytes-HEB, and considering the size of these circRNAs, we selected circPTN, which is derived from the *pleiotrophin* (*PTN*) gene, for further study. In gain- and loss-of-function experiments, we demonstrated that circPTN promoted the proliferation and increased the proportion of S-phase cells. Based on the bioinformatics prediction, we then hypothesized that circPTN may act by sponging miR-145-5p and miR-330-5p; we verified this using dual-luciferase reporter assays and biotin-labeled RNA pull-down experiments. In addition, we were surprised to find that circPTN was downregulated in serum-induced glioma stem cell (S15) compared with non-serum cultured glioma stem cell (G15), and we observed that circPTN promoted G15 to form tumor spheres and express stemness markers such as SOX2, SOX9, CD133, and Nestin via the sponging of miR-145-5p. However, the effect of circPTN on regulating self-renewal and the expression of stemness markers was ameliorated after the circPTN binding sites in miR-145-5p were mutated. We determined this similar effect in U87-GSC and U251-GSC. In conclusion, we propose that circPTN promotes the proliferation and genesis of glioma by sponging miR-145-5p/miR-330-5p.

## Methods and materials

### Tumor specimens and cell culture

Tumor specimens were collected from informed and consenting patients diagnosed with glioma, and the tissues were separated into several parts, which were subjected to RNA extraction and isolation of glioma stem cells (G15). Normal brain tissues were collected from patients without glioma who required the resection of brain tissues for other reasons, such as cerebral hemorrhage, cerebral contusion, and laceration. All methods were performed in accordance with the guideline approved by the Ethics Committee of Zhujiang Hospital. The primary glioma cells (G15) were isolated from a patient diagnosed with anaplastic oligodendroglioma [World Health Organization (WHO) grade III], according to the previously described procedure [29].

The human glioma cell line U251 was obtained from the Cell Bank of Type Culture Collection of the Chinese Academy of Sciences (Shanghai, China). The human glioma cell lines U87, LN18, A172 and U118 as well as 293 T cells were purchased from the American Type Tissue Culture Collection. Human astrocyte HEB was purchased from GuangZhou Jennio Biotech Co.,Ltd. The U87-Luc cell line was generated in our laboratory via transfection with a reporter gene encoding firefly luciferase as previous described [30]. These cells were cultured in Dulbecco modified Eagle medium (DMEM) containing 10% fetal bovine serum (FBS) (Gibco, CA, USA) and penicillin/streptomycin.

The G15 cells were cultured in DMEM/F12 with the addition of N2 and B27 (0.5×, Invitrogen Corporation, Carlsbad, CA, USA), bFGF and EGF (50 ng/mL, Sino Biological, Beijing, China), glutamine (1×, Invitrogen), and penicillin/streptomycin. For adherent culturing of G15 cells, the plates were covered with Laminin (Sigma, CA, USA). The S15 cells, which were the serum-differentiated form of G15 cells, were cultured in DMEM containing 10% FBS and penicillin/streptomycin. The U87-GSC and U251-GSC, which were the non-serum-undifferentiated form of U87 cells and U251 cells, were cultured in non-serum medium [31, 32]. For the examination of RNA stability, U251 cells were treated with actinomycin D at a concentration of 2 μg/mL.

### Transfection

SiRNAs and miRNA mimics were synthesized by GenePharma (Jiangsu, China). The oligonucleotide sequences were as follow:

#si-circPTN-1: 5′-TCAAGAATGCAGGCTCAAC-3′;

#sh-circPTN-1: 5′-TCAAGAATGCAGGCTCAAC-3′.

The template of circPTN or circPTN-145-mut with an artificial flanking sequence was synthesized and inserted into pcDNA 3.1, as previously described by Liang et al. [33]. For the transient transfection, siRNA, miRNA or plasmid were mixed Lipofectamine 2000 (Invitrogen) in OptiMEM (Gibco) to form complexes, and then transfected into the cells. The medium was changed after 6 h, and the RNA and protein extraction were performed at 48 h. For stable transfection, the lentivirus packaging of shRNA, circPTN, and circPTN-145-mut was prepared by GenePharma. Stably transfected cells were then selected with puromycin for 2 weeks. All sequences of siRNAs were listed in Additional file 1: Table S1.

### Quantitative RT-PCR and RNase R treatment

The total RNA was extracted using Trizol (Invitrogen), and 1000 ng of RNA were reverse-transcribed into cDNA using the PrimeScript™ RT Reagent Kit with gDNA Eraser (Takara Bio Inc., Shiga, Japan). Real-time polymerase chain reaction (RT-PCR) was performed using the SYBR® Premix Ex Taq™ II (Takara) and the Applied Biosystems 7500 Real-time PCR System (Applied Biosystems, Inc. Carlsbad, CA, USA). For treatment with RNase R (Epicentre, Madison, Wisconsin, USA), 1 μg of RNA was digested with 1 unit of RNase R at 37 °C for 10 min and 20 min. The gene expression data were normalized to *GADPH* and the expression of miR-145-5p/miR-330-5p was normalized to that of *U6*. The primer sequences used in this study are shown in Additional file 1: Table S1.

### Western blot

Cells were lysed in RIPA (CWBio, Beijing, China) containing protease inhibitors, on ice. The lysates were mixed with loading buffer and denatured at 100 °C for 10 min. The products were then subjected to sodium dodecylsulfate-polyacrilamide gel electrophoresis (SDS-PAGE) and transferred to polyvinylidene difluoride (PVDF) membranes (Millipore, Billerica, Massachusetts, USA). The membranes were incubated with primary antibodies [SOX2 (CST, 1:1000), SOX9 (Abcam, 1:5000), CD133 (Proteintech, 1:1000), Nestin (Proteintech, 1:1000), PTN (Abcam, 1:1500) and GADPH (Affinity, 1:1000)] and then the secondary antibody, which was conjugated to horseradish peroxidase (HRP;anti-rabbit IgG/anti-mouse IgG, CST, 1:5000). Immunoreative bands were visualized using Image Lab after the addition of luminol-based chemiluminescent substrate (ECL; Millipore). The immunobloting results were analyzed using Image Lab software.

### CCK-8 assay

We used a CCK-8 kit (Dojindo, Shanghai, China) to measure proliferation of U87 and U251 cells. A total of 1000 cells in a volume of 100 μL per well were cultured in five replicate wells in a 96-well plate in medium containing 10% FBS. Then, the CCK-8 reagent (10 μL) was added to 90 μL DMEM to generate a working solution, of which 100 μL was added per well and incubated for 1.5 h. We performed this assay at 0 h, 24 h, 48 h, 72 h, 96 h and 120 h.

### EdU assay

Cells were cultured in 96-well plate and treated with 100 μL of medium containing 20 μM EdU. After incubation at 37 °C, with 5% CO_2_ for 2 h, the cells were fixed with 4% paraformaldehyde for 30 min and incubated with 0.5% Triton-X-100 in PBS for 20 min. The nuclei were stained with Hoechst dye 33,342. The rate of proliferation was calculated according to the manufacturer’s instructions (KeyFluor488 Click-iT EdU Kit, keyGEN BioTECH, Jiangsu, China). Images of five randomly selected areas of each group were taken with a fluorescence microscope (Leica, Wetzlar, Germany).

### Cell cycle detection

Cells in logarithmic phase were dissociated with 0.25% trypsin, re-suspended in PBS and then fixed in ice-cold 70% ethyl alcohol overnight at 4 °C. Then, the cells were centrifuged at 1000 rpm for 5 min, re-suspended in 50 μL of RNase A and incubated at 37 °C for 30 min. Subsequently, 400 μL of propidium iodide (PI) was added to the suspension for 30 min, followed by detection with flow cytometry (BD bioscience, CA, USA).

### Tumor sphere formation assay

The G15 cells, U87-GSC and U251-GSC were harvested and re-suspended as single cells in a non-serum medium (as previously mention in Tumor specimens and cell culture). After accurate cell counting, 200 cells/well in 200 μL of non-serum medium were added to a 96 well plate, and each group was in 10 wells. The medium was change every 2 days. Image of five randomly selected regions of each group were taken with a fluorescence microscope (Leica, Wetzlar, Germany). The sphere percentage was calculated as the number of sphere/200.

### RNA pull-down

An RNA pull-down assay was performed as previously described [13, 20]. In brief, circPTN or antisense RNA was labeled with biotin and incubated with streptavidin beads (Thermo, CA, USA) at 4 °C overnight. The mixture was then centrifuged at 3000 rpm for 1 min and washed with Wash buffer I three times. A total of 2 × 10^7^ cells were lysed. The bead-biotin complex was added to the lysates and incubated at room temperature (RT) for 1 h. After washing with Wash buffer II, the RNA that was bound to the bead was captured and extracted with Trizol for the subsequent qPCR assay. The biotin-labeling of circPTN or antisense RNA was performed using Biotin RNA labeling Mix (Roche, Mannhei, Germany).

### Immunofluorescence

Cells were washed with PBS and fixed in 4% paraformaldehyde for 20 min at RT. The fixed cells were then incubated with 0.5% Triton-X-100 in PBS for 5 min at RT, then blocked with 5% BSA in PBS for 1 h at RT. Next, the cells were incubated with the primary antibodies against SOX2 (CST, 1:200), SOX9 (Abcam, 1:200), and CD133 (Proteintech, 1:200) at 4 °C overnight. Then, the cells were incubated with the fluorochrome-conjugated secondary antibody (CST, 1:200) as well as Nestin primary antibody conjugated to cy3 (Millipore, 1:200) at RT for 1 h. The nuclei were stained in 4,6-diamino-2-phenylindole (DAPI) for 15 min at RT. Images were captured using a fluorescence microscope (Zeiss).

### Fluorescence in situ hybridization

A cy3-labeled probe for detecting circPTN and FAM-labeled probes for detecting miR-145-5p/miR-330-5p were synthesized by GenePharma. Cells were cultured in 10% FBS, fixed by incubation with 4% paraformaldehyde for 20 min at RT, and then washed with PBS. The fixed cells were incubated with 0.5% Triton-X-100 in PBS for 5 min at RT. Subsequently, cells were incubated with pre-hybridization buffer for 30 min at 37 °C prior to the addition of probes in Hybridization buffer at 37 °C overnight. Next, the cover liquid was removed, and the cells were washed with 4× saline-sodium citrate (SSC), 2× SSC and 1× SSC for 3 × 5 min at 42 °C. The nuclei were stained with DAPI for 10 min at RT. The pre-hybridization buffer and hybridization buffer were obtained from the Ribo™ Fluorescent In Situ Hybridization Kit (Riobo biotech, Guangzhou, China). The sequences of probes are shown in Additional file 1: Table S1.

### Dual-luciferase reporter assay

The sequence of circPTN and the corresponding miR-145-5p/miR-330-5p mutants were cloned and inserted into the 3’UTR of psiCHECK-2 plasmid (Searching Biotechnology). In six-well plates, 293 T cells were cultured to approximately 70% confluence and then co-transfected with either wild type or mutant luciferase reporter vector (2 μg) and either mimic miRNAs or negative control (NC) (2 μg). After 48 h, luciferase activity was measured and normalized to the activity of Rluc.

### Intracranial xenograft

The method for establishing the intracranial xenograft model was previously described [30]. Briefly, 2 weeks after lentiviral transfection, 2 × 10^5^ U87-luc cells were injected stereotactically into the right hemicerebrum of 4- to 6- week-old female nude mice (*n* = 4, BALB/c-nu, Guangdong Medical Laboratory Animal Center, China). Tumor growth was monitored using an in vivo imaging system (IVIS Lumina II, Caliper, USA) after an intraperitoneal injection of luciferase substrate-D-luciferin (YEASEN, Shanghai, China). The tumors were excised on day 15 after the injection. A similar intracranial xenograft was established for evaluating survival time of nude mice. The observations continued past day 15 until the last mouse died naturally.

### Statistical analysis

Statistical analyses were performed using SPSS IBM 20.0. Statistical significance was determined by the *t-*test or ANOVA and the Mann-Whitney test (*p* < 0.05). Kaplan-Meier survival analysis and log-rank tests were used to analyze the difference in survival.

## Results

### circPTN is upregulated in glioma tissues and glioma cell lines compared with NBT and HEB cells

**Fig. 1:**
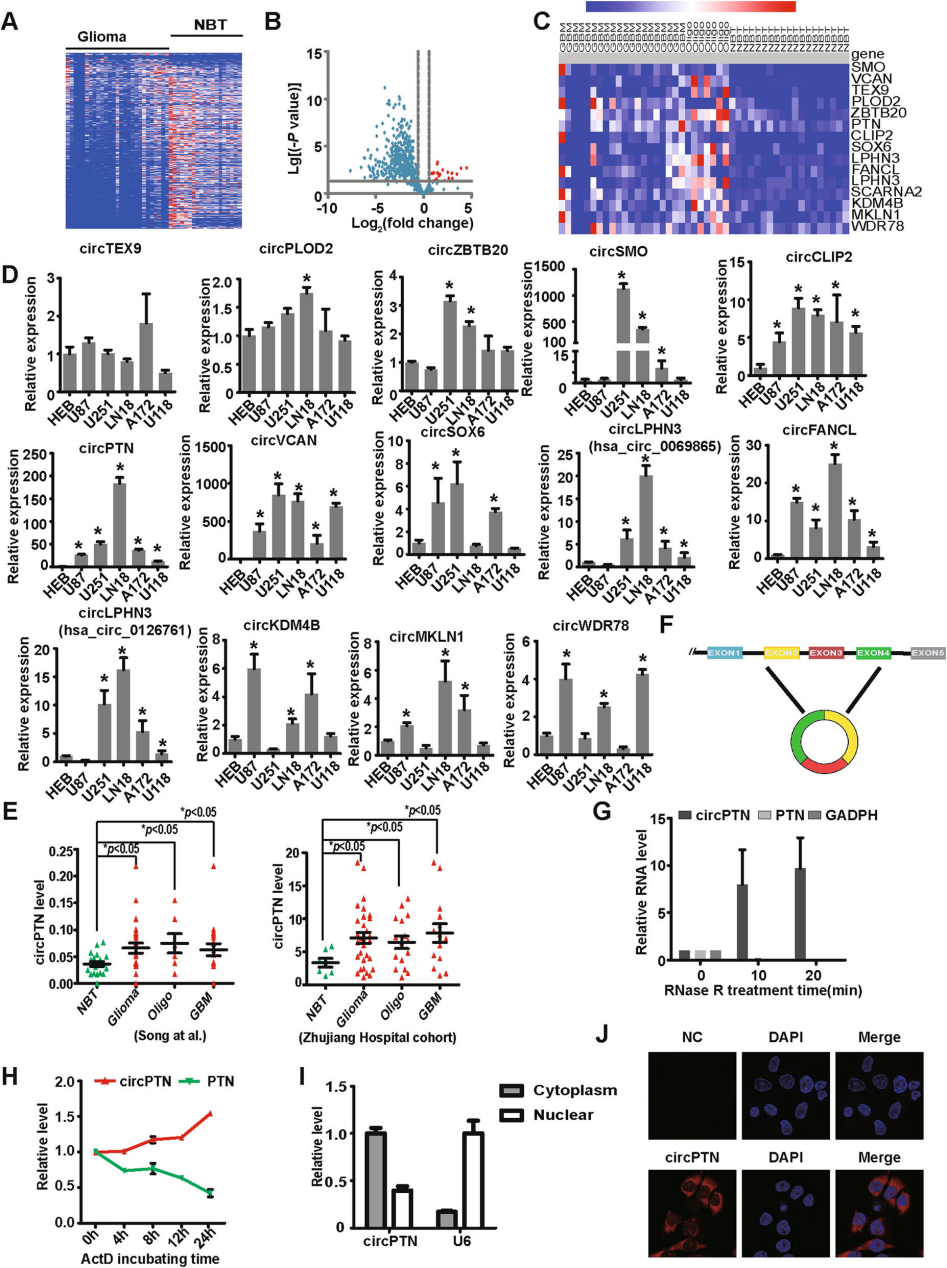
circPTN was upregulated in glioma tissues and glioma cell lines. **a**. The heat map of circRNAs in 27 glioma tissues vs. 19 NBT. **b**. The volcano plot of circRNAs expressed in glioma tissues vs. NBT; red plots represent circRNAs, which were at least 1.5-fold higher expression in glioma tissues than NBT (*p < 0.05). **c**. The heat map of 15 circRNAs, which were significantly upregulated in glioma tissues than NBT (> 1.5 fold, *p < 0.05). **d**. CircRNA expression in glioma cell lines and astrocyte-HEB cells. Compared with that in HEB cells, circCLIP2, circFANCL and circPTN were significantly upregulated in all glioma cell lines. (*n* = 3, *p < 0.05, *ANOVA*). **e**. circPTN was significantly upregulated in glioma tissues compared with NBT in both Song et al. cohort and Zhujiang Hospital cohort (30 glioma tissues vs. 7 NBT, *p < 0.05, *t test*); **f**. Schematic illustration showed that the circularization of *PTN* exons 2–4 formed circPTN. **g**. circPTN was resistant to RNase R treatment, whereas linear RNA-PTN and GADPH were not. (*n* = 3, mean ± SEM). **h**. qPCR for the abundance of circPTN and PTN in U251 cells treated with actinomycin D (2 μg/mL) at indicated time points. (*n* = 3, mean ± SEM). **i**. Nuclear-cytoplasm separation qPCR data suggested that circPTN was primarily localized in the cytoplasm. U6 almost located in the nucleus. (n = 3, mean ± SEM). **j**. By performing FISH, the images showed that circPTN existed in both cytoplasm and nucleus, but primarily localized in the cytoplasm in U251 cells; scale bar

We analyzed 572 highly expressed circRNAs in glioma and NBT, and we detected a lower expression of circRNAs in glioma tissues compared with NBT overall (Fig. 1a-b). However, we identified 15 circRNAs that were highly expressed in glioma tissues compared with NBT (> 1.5 fold, *P* < 0.05; Fig. 1c). Due to circSCARNA2 was not annotated by circBase, we measured the expressions of fourteen circRNAs (circTEX9-hsa_circ_0000603, circPLOD2-hsa_circ_0141963, circZBTB20-hsa_circ_0005332, circSMO-hsa_circ_0001742, circCLIP2-hsa_circ_0002755, and circPTN-hsa_circ_0003949, circSOX6- hsa_circ_0095454, circLPHN3- hsa_circ_0069865, circFANCL- hsa_circ_0001009, circLPHN3- hsa_circ_0126761, circKDM4B- hsa_circ_0002926, circMKLN1- hsa_circ_0001747 and circWDR78- hsa_circ_0006677) in glioma cell lines compared with HEB cells. The results demonstrated that circCLIP2, circVCAN, circFANCL and circPTN were significantly upregulated in all glioma cell lines (Fig. 1d). Considering that circVCAN at 5262 bp [34] was too large to be circularized for in vitro experiments, and given that circPTN expression compared with circCLIP2 and circFANCL, was much higher in glioma cell lines than in HEB, we decided to focus on circPTN. From cohort of Song et al., we observed that circPTN was significantly upregulated in glioma tissues compared with NBT but there was no difference between oligodendroglioma and GBM. We confirmed that circPTN was significantly upregulated in glioma tissues compared with NBT in our cohort (7 NBT vs 30 glioma tissues; Fig. 1e).

CircPTN originates from exons 2 to 4 of pleiotrophin (*PTN)* gene, and its mature length after splicing is 452 bp [34] (Fig. 1f). A previous study reported that circRNAs are resistant to RNase R and more stable than linear RNA. [6]. By performing qRT-PCR after treatment with RNase R in U251 cells, we demonstrated that circPTN was resistant to RNase R, whereas linear RNAs including PTN and GADPH were digested by RNase R (Fig. 1g). Next, we determined that circPTN was more stable than PTN after treatment with actinomycin D in U251 cells (Fig. 1h). We also determined that circPTN is primarily located in cytoplasm by performing fluorescence in situ hybridization (FISH) experiments and nuclear and cytoplasmic separation qRT-PCR in U251 cells (Fig. 1i-j). These indicate that circPTN may be an oncogenic factor and may act through sponging miRNAs, given its location in cytoplasm.

### circPTN promotes glioma proliferation in vitro and in vivo

**Fig. 2:**
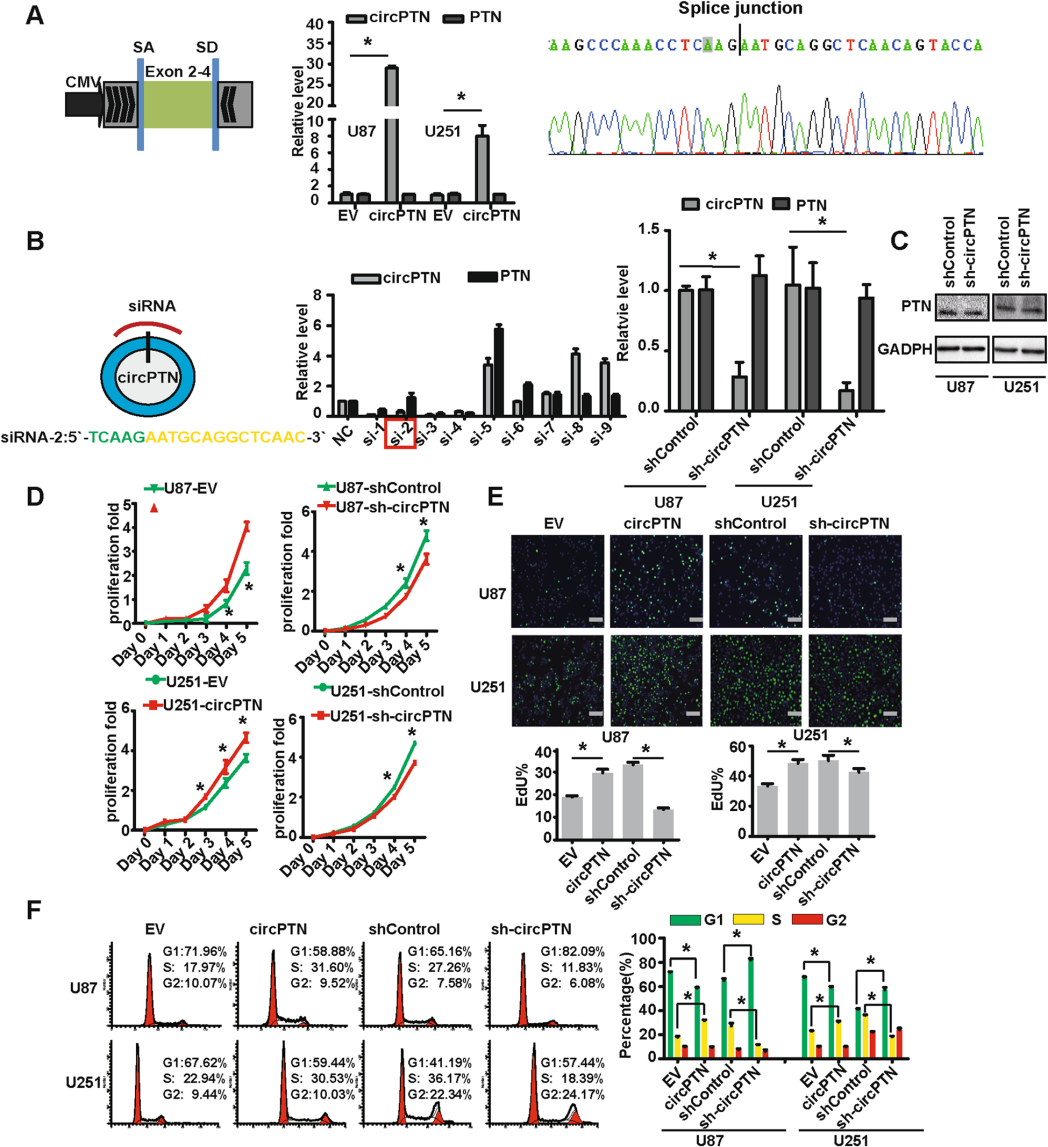
circPTN promoted glioma growth in vitro*.* **a**. Left: Schematic illustration for *minigene* for circularizing circPTN in vitro: exons 2–4 of the PTN gene were cloned between splicing acceptor (SA) and splicing donor (SD) sequences with upstream and downstream flanking inverted repeat sequences; Middle: Stable overexpression for circPTN by lentiviral tranfection in U87 and U251 cells, *n* = 3, *p < 0.05, *t test*; Right: Sanger sequence indicated our overexpression system produced circPTN with correct junction. **b**. Left: Schematic illustration for siRNA specially targeted junction of circPTN; Middle: Nine siRNAs across the splice junction and one that can specifically target circPTN but not influence the linear spliced *PTN* product, (n = 3, mean ± SEM); Right: Stable interference system for circPTN by lentiviral transfection in U87 and U251 cells, n = 3,*p < 0.05, *t test*. **c**. Images of western blots between shControl and sh-circPTN in U87 and U251 cells. **d**. Results of CCK-8 assays indicated that circPTN significantly promoted proliferation of U87 and U251 cells. *n* = 3, *p < 0.05, *t test*. **e**. Upper: Results of EdU assays showed that circPTN significantly increased the percentage of EdU positive U87 and U251 cells; scale bar, 100 μm; Lower: Analyses of EdU assays results indicated circPTN promoted proliferation. *n* = 5, *p < 0.05, *t test*. **f**. Left: Cell cycle showed that circPTN increased proportion of S phase cells in U87 and U251 cells; Right: Analyses of cell cycle results indicated that circPTN significantly promoted shift of G1 → S, *n* = 3, *p < 0.05, *t test*

To circularize circPTN in vitro, we constructed a *minigene* vector [33] and confirmed that this vector was correctly circularized by Sanger sequencing (Fig. 2a). Moreover, we designed nine siRNAs across the splice junction and identified one siRNA that specifically targeted circPTN but did not influence the linear spliced *PTN* product. We succeeded in establishing stable overexpression and interference system for circPTN by lentiviral transfection in U87 and U251 cells (Fig. 2b). Besides, the protein level of PTN did not altered in U87 and U251 cells after transfections of sh-circPTN (Fig. 2c). By performing CCK-8 and EdU assays, we demonstrated that overexpression of circPTN significantly promoted the proliferation of U87 and U251 cells, whereas the interference of circPTN inhibited the proliferation of U87 and U251 cells. Utilizing flow cytometry, we determined overexpression of circPTN promoted the transition of G1-S phase in U87 and U251 cells, and we observed the opposite trend with the interference of circPTN (Fig. 2d-f). These results indicate that circPTN promotes glioma proliferation in vitro.

**Fig. 3:**
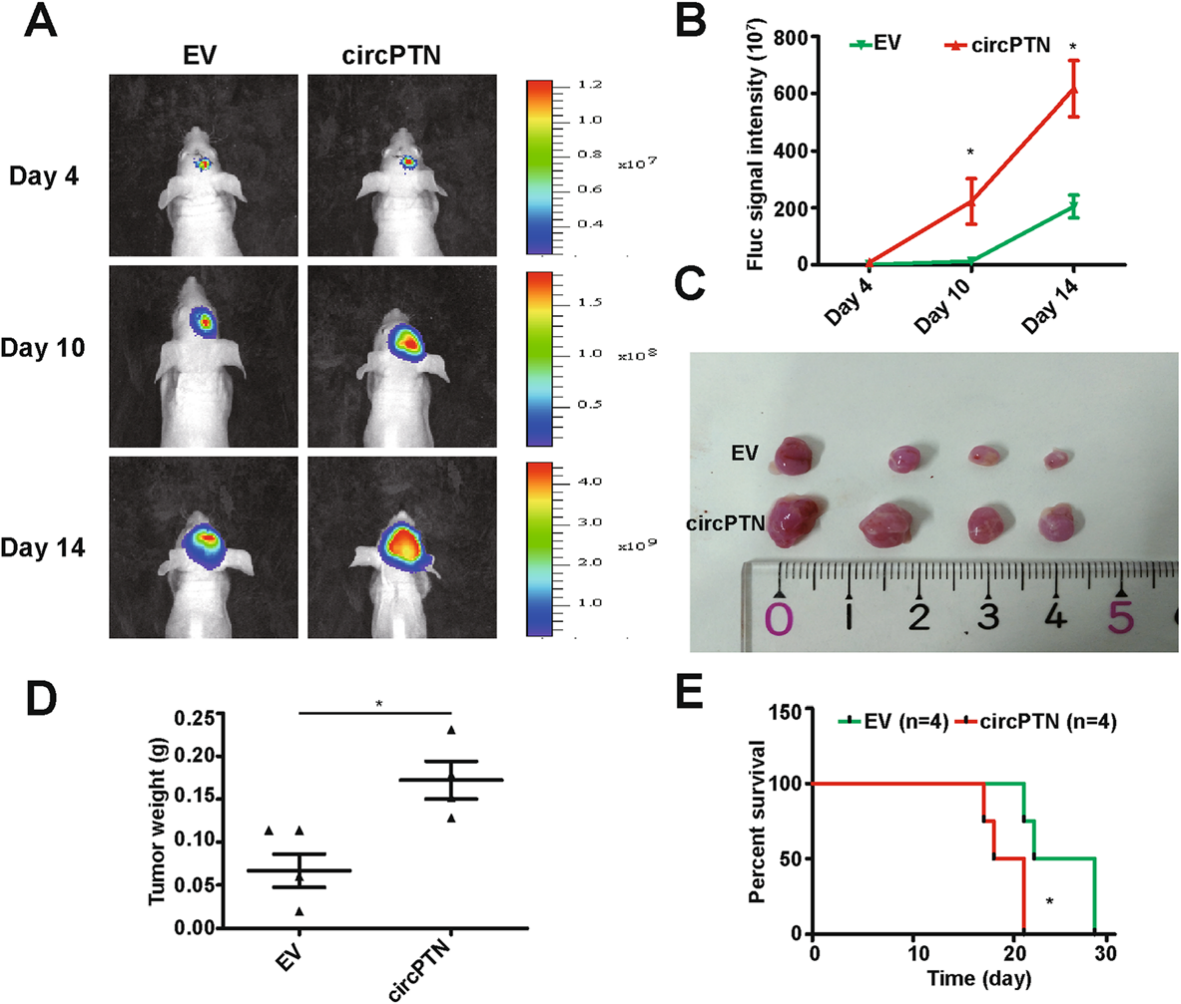
circPTN promoted glioma growth in vivo. **a**. Images from intracranial xenograft of BALB/c-nu after injection of D-luciferin under in-vivo imaging system; **b**. Results showed that the growth rate was significantly increased in group circPTN compared with group EV, *n* = 4, *p < 0.05, *t test*; **c**. Tumors that were harvested in day 15; **d**. Results showed that the tumor weight was significantly increased in group circPTN compared with group EV, n = 4, *p < 0.05, *t*-test; **e**. Mice in group circPTN had shorter survival time than mice in group EV by Kaplan-Meier survival analysis, n = 4, *p < 0.05, log-rank test

After demonstrating the impact of circPTN in vitro*,* we aimed to investigate whether circPTN influences the biological behavior of tumors in vivo. Therefore, we used stably lentiviral transfected U87-luc-EV and U87-luc-circPTN cells to establish a nude mouse intracranial xenograft model. Our results demonstrated that the tumor growth rate and tumor weights were significantly increased in the circPTN group compared with the EV group (Fig. 3a-d). Moreover, we established another nude mouse intracranial xenograft model similar to the former and found that mice in group circPTN had shorter survival time than mice in group EV (Fig. 3e). These results suggested that circPTN promoted tumor growth in vivo.

### circPTN sponges miR-145-5p and miR-330-5p and accelerates their degradation

**Fig. 4:**
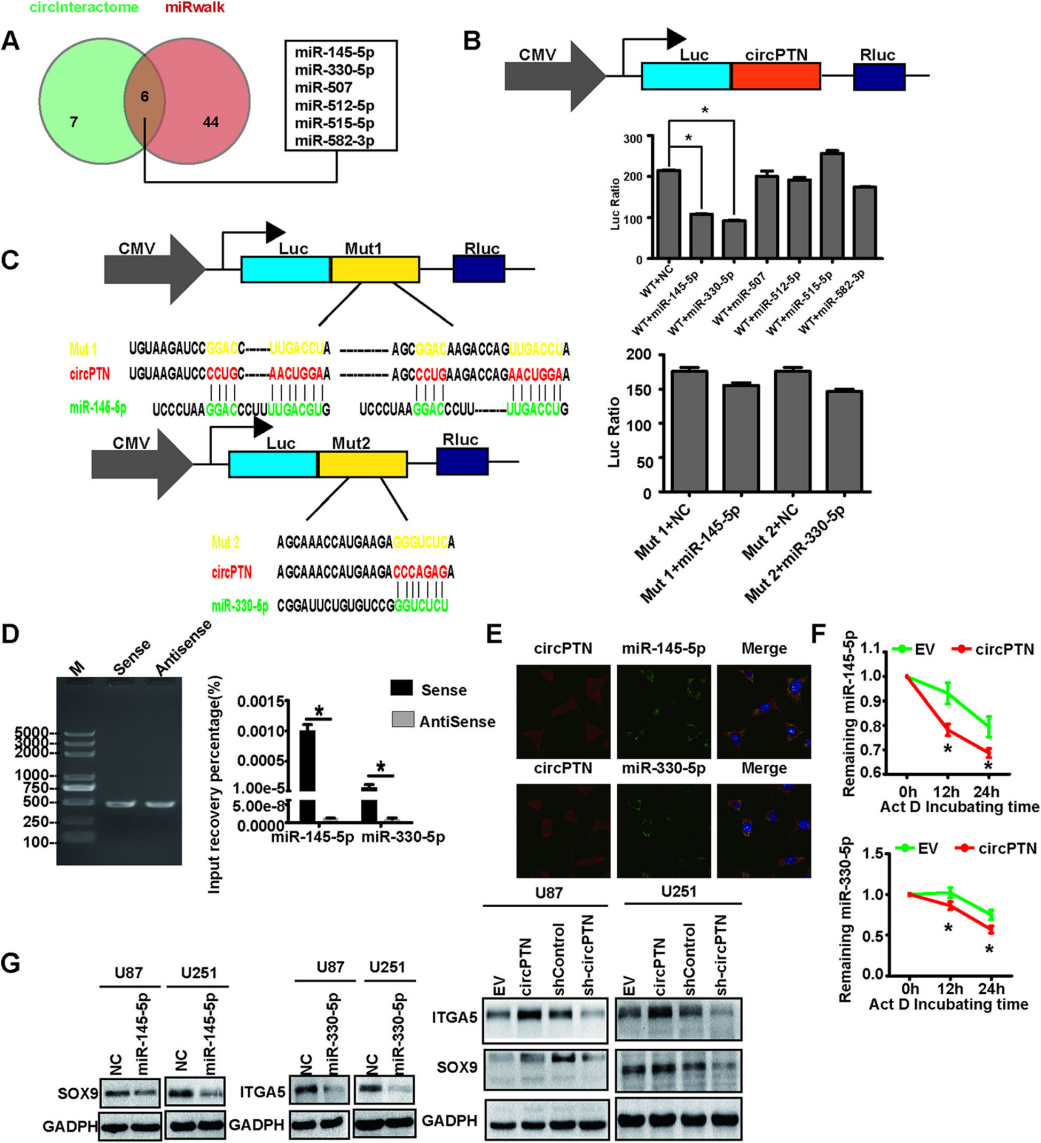
circPTN sponges miR-145-5p and miR-330-5p. **a**. Venn diagram for circInteractome and *miRWalk* of predicting miRNAs sponged by circPTN. **b.** Dual-luciferase reporter assay showed that co-transfection of WT and mimic miR-145-5p or mimic miR-330-5p markedly decreased luciferase activity in 293T cells, n = 3, *p < 0.05, *t test*. **c.** Results of dual-luciferase reporter assay indicated that luciferase activity did not change in 293T cells when miR-145-5p or miR-330-5p binding sites in circPTN were mutated, n = 3, *p < 0.05, *t test*. **d.** Left: Biotin-labeled circPTN or anti-sense RNA in agarose gel; Right: Histogram of qPCR of RNA pull-down assay. Compared with group anti-sense, miR-145-5p and miR-330-5p in group circPTN were significantly abundant, n = 3, *p < 0.05, *t test*. **e.** Images of Cy3-labeled probe for circPTN and FAM-labeled probes for miR-145-5p/miR-330-5p indicated that circPTN and miR-145-5p/miR-330-5p co-localized in the cytoplasm; scale bar, 20 μm. **f.** U251 cells stably transfected EV or circPTN were treated with 2 μg/mL actinomycin D or DMSO at indicated time point; the results suggested that compared with group EV, miR-145-5p and miR-330-5p were significantly less than the level observed in circPTN group, n = 3, *p < 0.05, *t test*. **g.** circPTN could rescue the downregulation of SOX9 and ITGA5 by miR-145-5p and miR-330-5p, n = 3, *p < 0.05, *t test*

Next, we aimed to investigate how circPTN promotes glioma proliferation. Previous studies have reported that circRNA can function as a miRNA sponge. Considering that circPTN is primarily localized to the cytoplasm, we hypothesized that circPTN may regulate the biological behavior of tumors by sponging miRNAs. We utilized *circInteractome* [35] and *miRWalk* to predict miRNAs that would likely be sponged by circPTN, and both databases identified six such miRNAs (Fig. 4a). To confirm this prediction, we constructed a dual-luciferase reporter system by inserting the sequence of circPTN into the 3′ UTR of the psiCHECK2 plasmid (wild type, WT). The results showed that, when co-transfected with WT and NC or miRNAs, the mimic miR-145-5p and mimic miR-330-5p significantly decreased luciferase activity (Fig. 4b). After that, we cloned two mutated sequences into 3′ UTR of psiCHECK2 plasmid, which were binding sites for miR-145-5p and miR-330-5p in circPTN mutated, respectively. However, we did not observe obvious change in luciferase activity after co-transfection with Mut 1/Mut 2 and the corresponding miRNA mimic (Fig. 4c). Moreover, we performed an RNA pull-down assay to investigate whether circPTN directly interacts with miR-145-5p/miR-330-5p. Biotin-labeled circPTN was incubated with total RNA extracted from U251 cells; the anti-sense sequence of biotin-labeled circPTN served as a control. Magnetic bead-labeled streptavidin was used to capture the biotin, and the captured product was subjected to qPCR. The results demonstrated that compared with antisense group, miR-145-5p and miR-330-5p were significantly enriched in the circPTN group (Fig. 4d). In addition, we performed FISH and confirmed that circPTN co-localized with miR-145-5p and miR-330-5p in the cytoplasm (Fig. 4e).

After verifying that circPTN has the capacity to sponge miR-145-5p/miR-330-5p, we questioned whether circPTN can influence the degradation of miR-145-5p/miR-330-5p. U251 cells stably transfected with EV or circPTN were treated with actinomycin D or Dimethyl sulfoxide (DMSO). At time points of 0 h, 12 h and 24 h after treatment, total RNA was extracted and the abundance of miR-145-5p/miR-330-5p was measured. The results suggested that the levels of miR-145-5p and miR-330-5p were significantly decreased in the circPTN group compared with the empty vector (EV) group (Fig. 4f). SOX9 and ITGA5 are known as downstream targets of miR-145-5p and miR-330-5p [36, 37], respectively, which we confirmed by measuring the protein level of SOX9 and ITGA5 after transfection with miR-145-5p/miR-330-5p. To further confirm that circPTN was sponging miR-145-5p/miR-330-5p, we also measured the protein level of SOX9 and ITGA5 after overexpression or knockdown of circPTN. The result showed that circPTN rescued the downregulation of SOX9 and ITGA5 by miR-145-5p and miR-330-5p, respectively (Fig. 4g), suggesting that circPTN could sponge both miR-145-5p and miR-330-5p and accelerate their degradation. These results indicate that circPTN can sponge miR-145-5p and miR-330-5p.

### MiR-145-5p and miR-330-5p are downregulated in glioma and inhibit glioma cells proliferation; this inhibition of proliferation can be rescued by circPTN

**Fig. 5:**
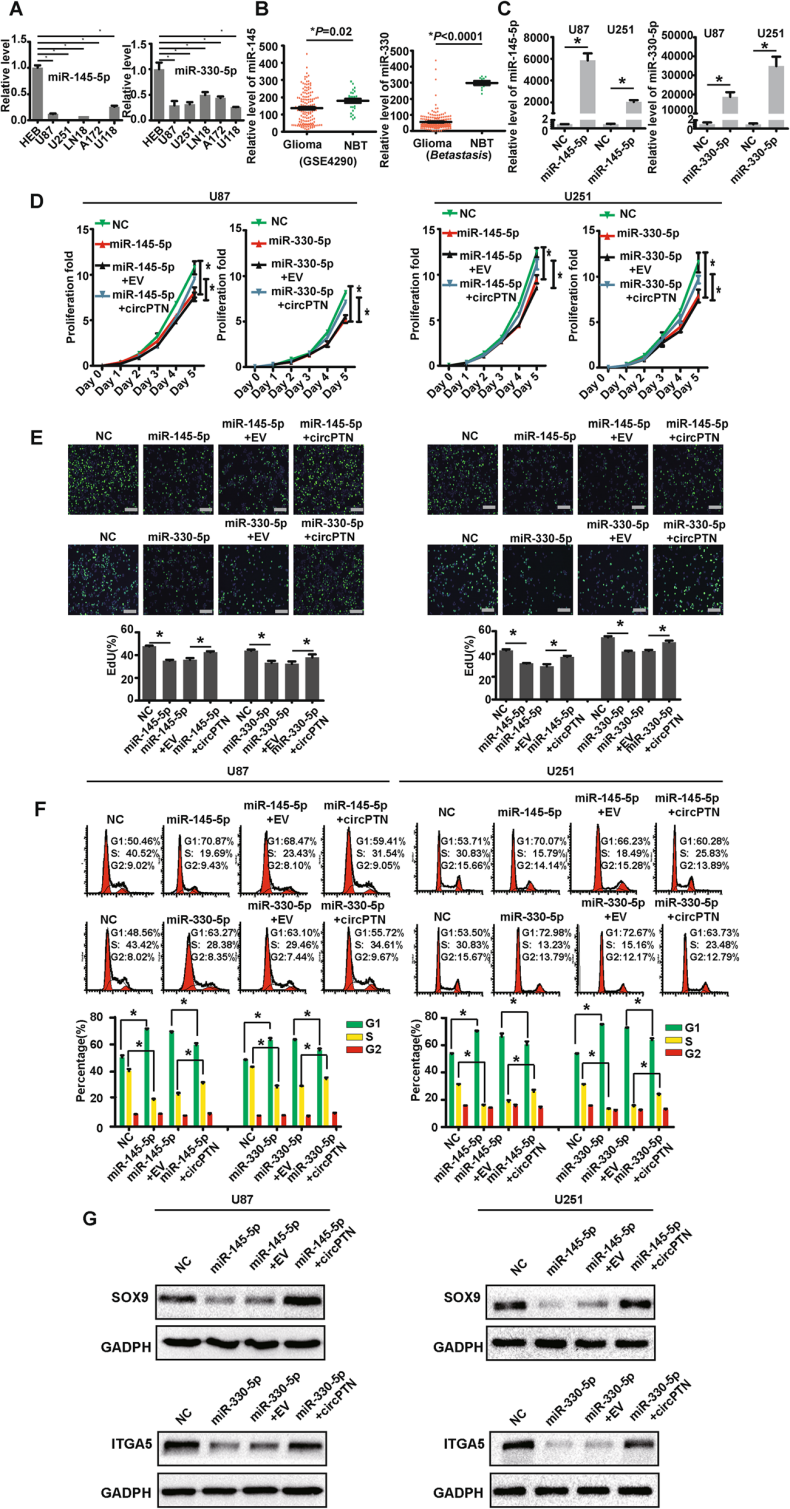
miR-145-5p and miR-330-5p are downregulated in glioma, inhibit glioma cells proliferation, and the inhibition of proliferation can be rescued by circPTN. **a**. miR-145-5p and miR-330-5p were significantly downregulated in glioma cell lines compared with HEB cells, *n* = 3, *p < 0.05, *ANOVA*. **b**. Data from *GEO datasets* (GSE4290) and *Betastasis* indicated that miR-145 and miR-330 were significantly downregulated in glioma tissues compared with NBT, respectively, *p < 0.05, *t test*. **c**. Stable overexpression system of miR-145-5p and miR-330-5p by lentiviral transfections in U87 and U251 cells, n = 3, *p < 0.05, *t test*. **d**. Results of CCK-8 assays suggested that miR-145-5p and miR-330-5p significantly inhibited proliferation of U87 and U251 cells, and enforcing expression of circPTN could rescue the inhibition of proliferation by miR-145-5p and miR-330-5p, *n* = 3, *p < 0.05, *t test*. **e**. Upper: Results of EdU assays showed that miR-145-5p and miR-330-5p significantly decreased the percentage of EdU-positive U87 and U251 cells, and enforcing expression of circPTN could rescue the decrease; scale bar, 100 μm. Lower: Analyses of EdU assays, n = 5, *p < 0.05, *t test*. **f**. Left: Cell cycle showed that miR-145-5p and miR-330-5p significantly arrest the shift of G1 → S, enforcing expression of circPTN could rescue the arrest in U87 and U251 cells; Right: Analyses of cell cycle results, n = 3, *p < 0.05, *t test*. **g**. The inhibition of SOX9/ITGA5 expression by miR-145-5p/miR-330-5p could be rescued by enforcing circPTN expression in U87 and U251 cells

After confirming that circPTN could sponge miR-145-5p and miR-330-5p, we sought to determine the role of these miRNAs in glioma. Although previous studies have reported that miR-145-5p and miR-330-5p are tumor suppressive factors that inhibit cell proliferation [36, 37], we aimed to confirm this effect in our experimental system. First, we measured the expression of miR-145-5p and miR-330-5p in glioma cell lines and HEB cells; as expected, miR-145-5p and miR-330-5p were significantly downregulated in the glioma cell lines compared with HEB cells (Fig. 5a). Moreover, we analyzed the data from *GEO datasets* and *Betastasis*, and we determined that miR-145 and miR-330 were downregulated in glioma tissues compared with NBT (Fig. 5b). These results indicate that miR-145-5p and miR-330-5p are tumor-suppressive factors.

Next, we succeeded in establishing a stable overexpression system of miR-145-5p and miR-330-5p through the lentiviral transfection of U87 and U251 cells (Fig. 5c). By performing CCK-8 and EdU assays in U87 and U251 cells, we determined that stable transfections of mimic miR-145-5p and mimic miR-330-5p inhibited glioma cell proliferation and contributed to the G1 → S phase cell cycle arrest. Importantly, we found that the inhibition of cell proliferation and the G1 → S shift by miR-145-5p and miR-330-5p in U87 and U251 cells could be rescued by increasing the expression of circPTN (Fig. 5d-f). Besides, we demonstrated that the downregulation of SOX9/ITGA5 by miR-145-5p/miR-330-5p could be rescued by overexpression of circPTN (Fig. 5g). These results suggeste that circPTN sponges miR-145-5p/miR-330-5p to promote the proliferation of glioma.

### circPTN promotes the stemness of glioma stem cells by sponging miR-145-5p

We found that circPTN sponges miR-145-5p, and miR-145-5p has been previously described as being associated with the regulation of stemness or self-renewal [38–40]. In addition, growing evidence suggests that glioma-stem cell (GSCs), clusters of cancer cells, possess the capacity for self-renewal and may be the origin of tumorigenesis [41]. We therefore hypothesized that circPTN may influence self-renewal.

**Fig. 6:**
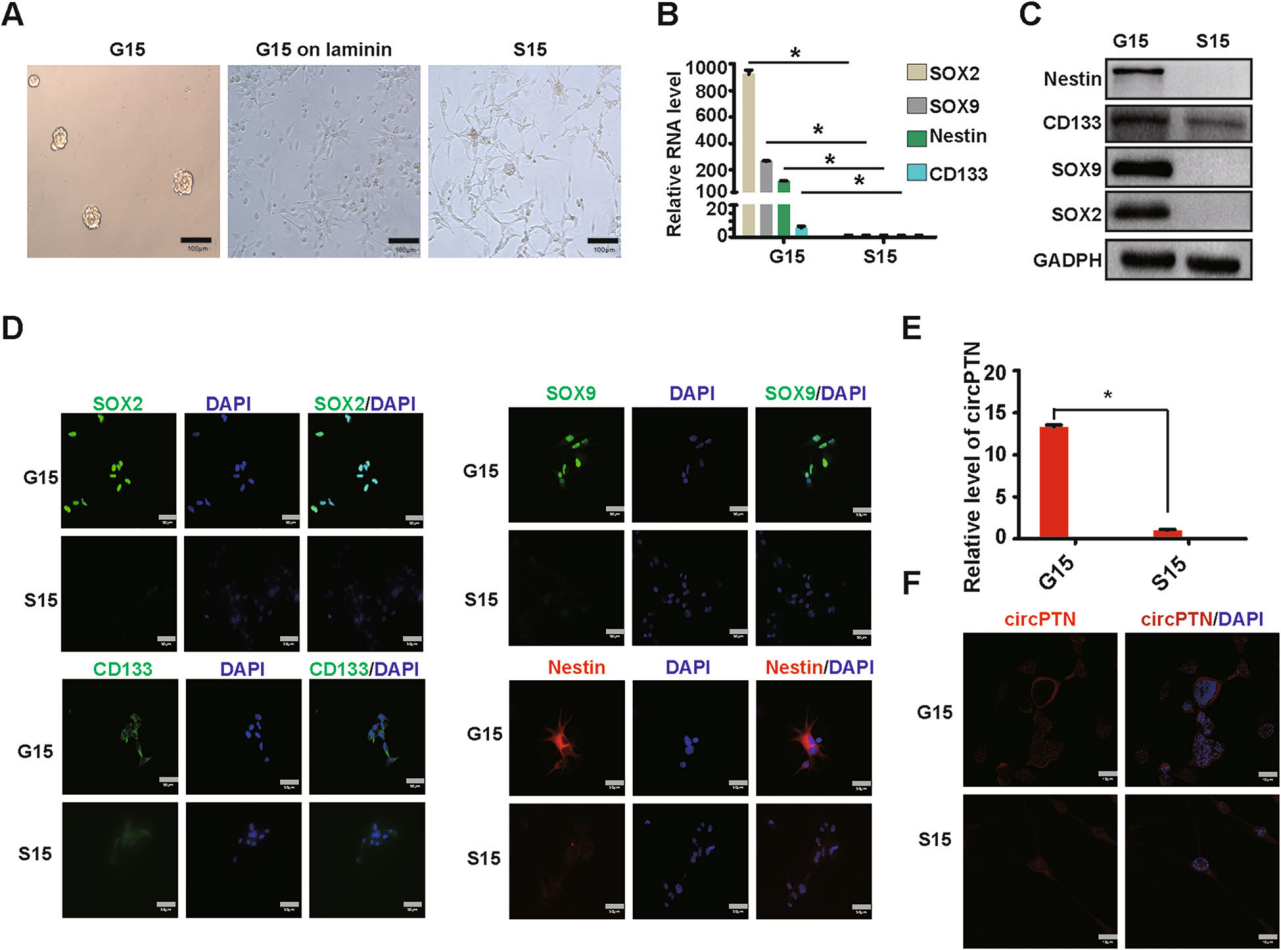
The expression of stemness markers in G15 cells and S15 cells. **a**. Left: G15 cells in suspension cultivation; Middle: G15 cells was cultivated on laminin and presented morphologically as short spindles; Right: S15 cells in adherent cultivation exhibited longer spindle morphology; scale bar, 100 μm. **b**. qPCR results of stemness markers in G15 cells and S15 cells, suggested that stemness markers significantly upregulated in G15 cells compared with that in S15 cells, *n* = 3, *p < 0.05, *t test*. **c**. Results of western blot assay showed stemness markers were higher in G15 cells than in S15 cells. **d**. Images of immunofluorescence for stemness markers in G15 cells and S15 cells. **e**. The expression of circPTN was significantly higher in G15 cells that in S15 cells, *n* = 3, *p < 0.05, *t test*. **f**. Cy3-labeled probe for circPTN in G15 cells and S15 cells by performing FISH. Images showed that circPTN majorly localized in the cytoplasm in G15 cells and S15 cells; scale bar, 10 μm

G15 cells, glioma stem cells derived from an anaplastic oligodendroglioma (WHO grade III), was cultured in non-serum conditions. G15 cells could form tumor spheres in suspension cultivation and presented morphologically as short spindles when cultured on laminin-covered plates. S15 cells, which were derived from G15 cells after serum-induction, could not form tumor spheres, and they were able to be cultured in the adherent state, exhibiting longer spindle morphology (Fig. 6a). Next, we examined stemness markers, including Nestin, CD133, SOX2, and SOX9 in G15 and S15 cells. As expected, the levels of these markers were significantly higher in G15 cells compared with S15 cells (Fig. 6b-d). Importantly, the expression of circPTN in G15 cells was approximately 10-fold higher than that in S15 cells, suggesting that circPTN may play a role in regulating stemness (Fig. 6e). In addition, we used FISH to confirm that the majority of circPTN was localized to the cytoplasm in both G15 and S15 cells (Fig. 6f). These results suggest that circPTN may play a role in regulating self-renewal.

**Fig. 7:**
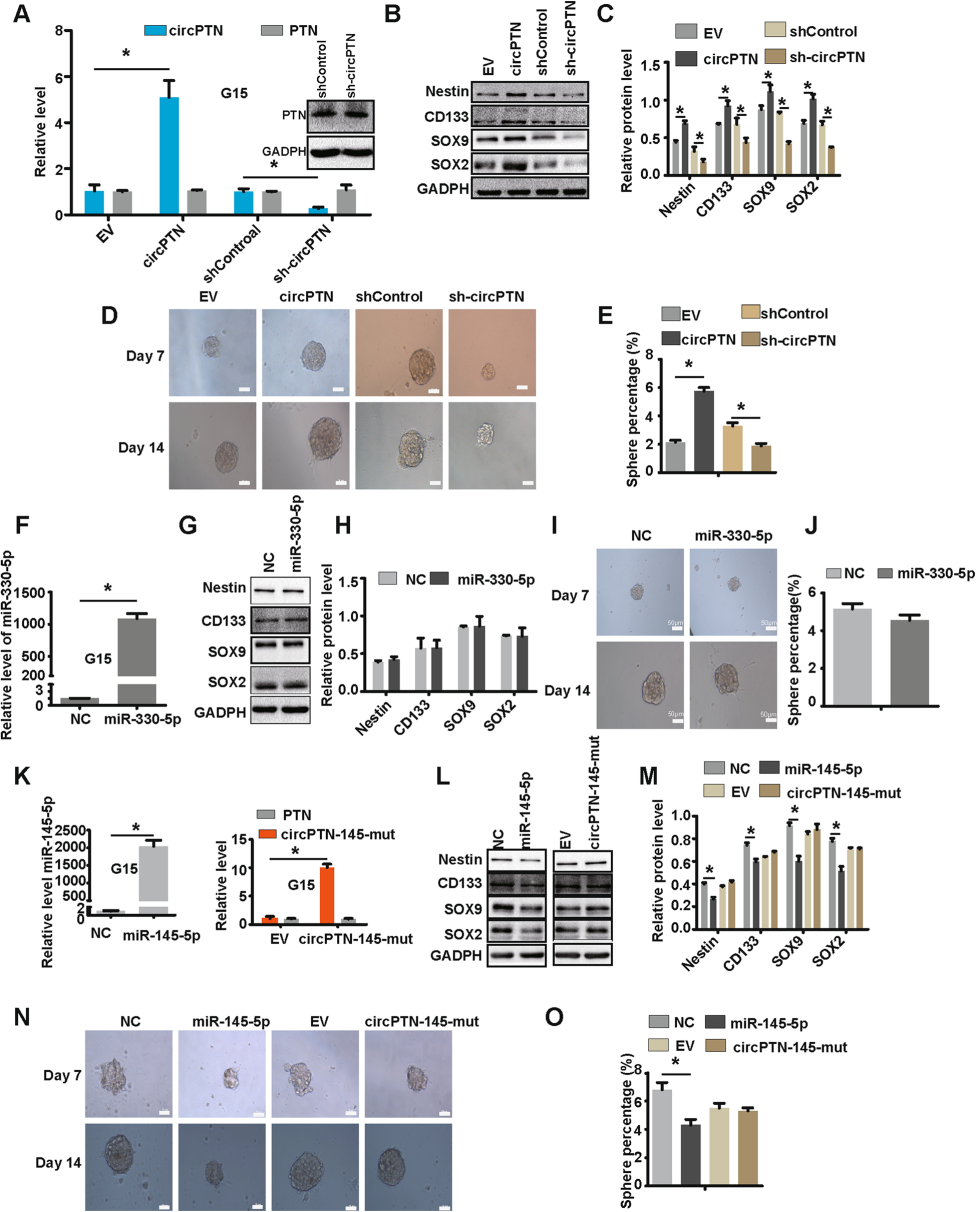
circPTN promotes GSCs self-renewal via sponging miR-145-5p. **a**. Outside: Stable overexpression and interference system for circPTN by lentiviral transfection in G15 cells, n = 3, *p < 0.05, *t test*; Inside: Images of western blot between shControl and sh-circPTN in G15 cells. **b**. Image of western blot assay of stemness markers. **c**. Analyses of results of western blot assay suggested that circPTN significantly upregulated protein level of stemness markers, n = 3, *p < 0.05, *t test*. **d**. Images of tumor sphere formation assay (200 cells/well); scale bar, 50 μm. **e**. Analyses of tumor sphere formation results indicated that circPTN significantly promoted self-renewal of G15 cells, *n* = 10, *p < 0.05, *t test*. **f**. Stable overexpression system of miR-330-5p by lentiviral transfection in G15 cells, n = 3, *p < 0.05, *t test*. G. Image of western blot assay of stemness markers; **h**. Analyses of results of western blot indicated that miR-330-5p did not influence the protein level of stemness markers, n = 3, *p < 0.05, *t test*. **i**. Images of tumor sphere formation assays in G15 cells (200 cell/well); scale bar, 50 μm; **j**. Analyses of tumor sphere formation results indicated that miR-330-5p did not influence self-renewal of G15 cells, n = 10,*p < 0.05, *t test*. **k**. Stable overexpression system of miR-145-5p and circPTN-145-mut by lentiviral transfection in G15 cells, n = 3, *p < 0.05, *t test*. **l.** Image of western blot assay of stemness markers. **m.** Analyses of results of western blot assay indicated that miR-145-5p significantly decreased the protein level of stemness markers, whereas circPTN-145-mut did not influence them, *n* = 3, *p < 0.05, *t test*. **n.** Images of tumor sphere formation assay in G15 cells (200 cell/well); scale bar, 50 μm. **o.** Analyses of tumor sphere formation results indicated that miR-145-5p significantly inhibited self-renewal of G15 cells, whereas circPTN-145-mut did not influence self-renewal, n = 10,*p < 0.05, *t test*

Consequently, after establishing a stable overexpression and interference system of circPTN through the lentiviral transfection in G15 cells (Fig. 7a), we analyzed the expression of stemness markers and determined that these markers were positively associated with circPTN levels (Fig. 7b-c). In addition, we performed a tumor sphere formation assay to investigate the influence of circPTN on self-renewal. The results showed that the sphere percentage was significantly increased in the circPTN group compared with the EV group. In contrast, after interfering with circPTN expression, the sphere percentage was significantly decreased (Fig. 7d-e). Since we had demonstrated that circPTN could sponge miR-145-5p and miR-330-5p, we hypothesized that circPTN may regulate self-renewal via sponging miR-145-5p and/or miR-330-5p. After establishing a stable overexpression system of mimic miR-330-5p through the lentiviral transfection of G15 cells (Fig. 7f), we performed a tumor sphere formation assay and measured the expression of stemness markers (Nestin, CD133, SOX9, and SOX2). However, we did not observe a significant difference in the tumor sphere percentage or the expression of stemness markers between the NC and mimic miR-330-5p (Fig. 7g-j). After establishing a stable overexpression system of mimic miR-145-5p through the lentiviral transfection of G15 cells (Fig. 7k), we determined that the sphere percentage and the levels of Nestin, CD133, SOX2, and SOX9, as well as tumor sphere percentage were markedly decreased in the mimic miR-145-5p group compared with the NC group (Fig. 7l-o). These results suggest that circPTN promotes GSCs self-renewal via sponging miR-145-5p. To confirm this hypothesis, we constructed the plasmid-circPTN-145-mut, which contained mutated binding sites for miR-145-5p in circPTN. After establishing the stable overexpression system of circPTN-145-mut (Fig. 7k), we performed a tumor sphere formation assay and measured the expression of stemness markers. The results indicated that there was no difference in sphere percentage between EV and circPTN-145-mut transfected cells, nor was there any change in the levels of stemness markers (Fig. 7l-o).

**Fig. 8:**
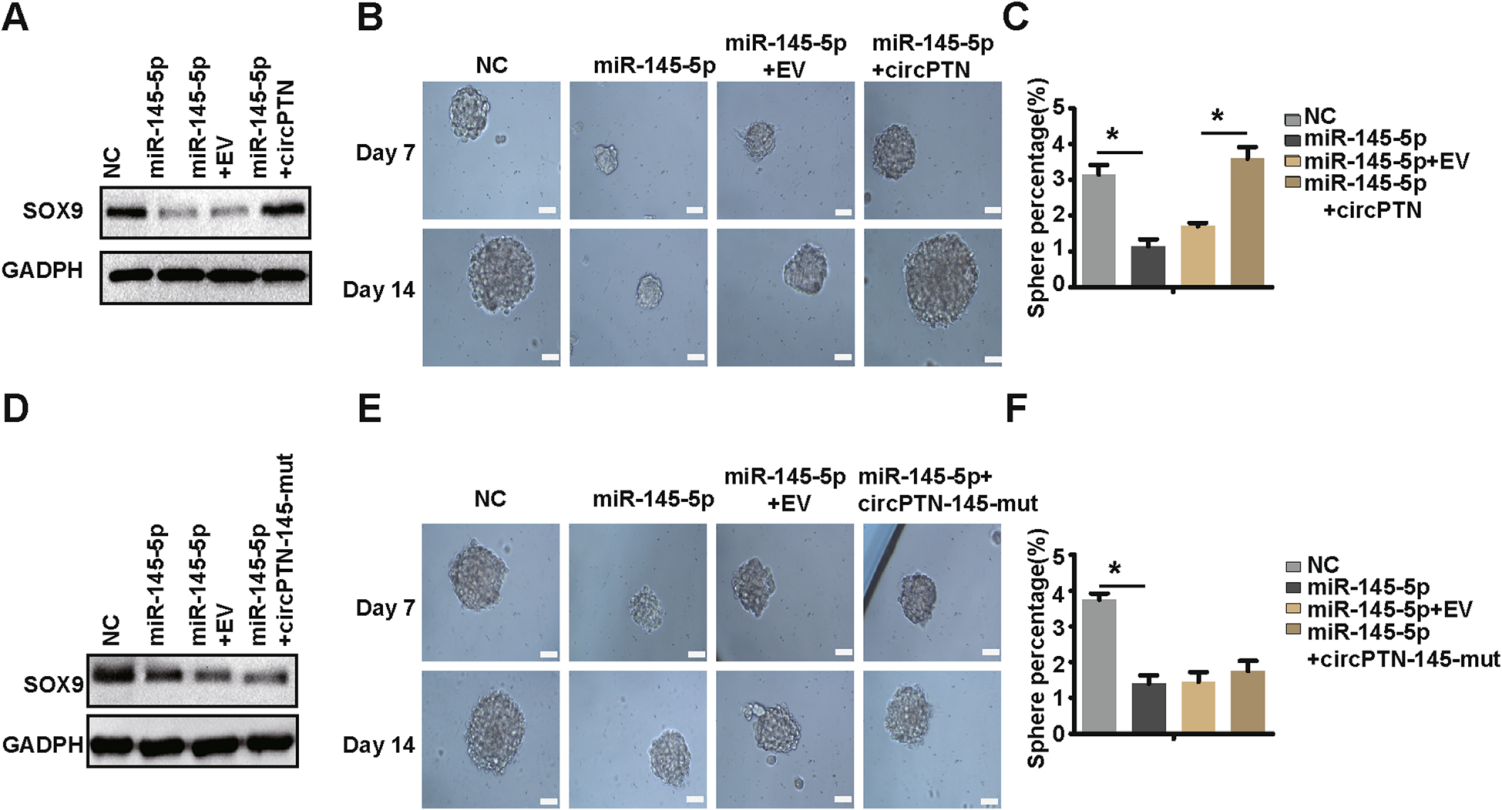
circPTN rescues the inhibition of GSCs self-renewal by miR-145-5p. **a.** Results of western blot assay showed that circPTN could rescue the inhibition of SOX9 expression by miR-145-5p. **b.** Images of tumor sphere formation assay (200 cells/well); scale bar, 50 μm. **c.** Analyses of tumor sphere formation results indicated that circPTN could rescue the inhibition of self-renewal of G15 cells by miR-145-5p, *n* = 10, *p < 0.05, *t test*. **d.** Results of western blot assay showed that circPTN-145-mut failed to rescue the inhibition of SOX9 expression by miR-145-5p.**e.** Images of tumor sphere formation assay (200 cells/well); scale bar, 50 μm. **f.** Analyses of tumor sphere formation results indicated that circPTN-145-mut failed to rescue the inhibition of self-renewal of G15 cells by miR-145-5p, *n* = 10, *p < 0.05, *t test*

Moreover, we demonstrated that the inhibition of self-renewal by miR-145-5p could be rescued by overexpression of circPTN (Fig. 8a-c), whereas overexpression of circPTN-145-mut failed to rescue the inhibition by miR-145-5p (Fig. 8d-f). After demonstrating the positive regulation of circPTN in G15 cells, we confirmed this regulation of circPTN in U87-GSC and U251 GSC, which were glioma stem cells derived from U87 cells and U251 cells, respectively (Additional file 2: Figure S1, Additional file 3: Figure S2, Additional file 4: Figure S3). Taken together, these results suggest that circPTN regulates self-renewal via sponging miR-145-5p.

## Discussion

CircRNA was discovered over 30 years ago, but it has only recently attracted attention since Memczak and Hansen [4, 5] reported that CDR1as has more than 70 binding sites for miR-7 and acts as a miR-7 sponge. Many studies have reported that, in addition to its action as a miRNA sponge, circRNA may interact with RNA-binding proteins [20–22], regulate transcription [23], and translate into proteins [24, 25].

In this study, we analyzed the sequencing data from normal brain tissue and glioma tissues reported by Song et al. [28]. After screening the data based on the expression in HEB cells and glioma cell lines, we chose circPTN as our experimental subject. By measuring circPTN expression in our cohort of NBT and glioma tissues, we confirmed that circPTN was highly expressed in glioma tissues compared with normal brain tissues. As with most circRNAs, circPTN was resistant to RNase R and was more stable than linear RNA after treatment with actinomycin D. Using FISH and nuclear-cytoplasmic separation qPCR, we determined that circPTN was primarily localized to the cytoplasm, which suggested that circPTN may act by sponging miRNA. In gain- and loss-of-function experiments, we observed that circPTN promoted proliferation and increased the proportion of S-phase cell in U251 and U87 cells. Next, based on the predictions of *CircInteractome* and *miRwalk*, we hypothesized that miR-145-5p and miR-330-5p may be circPTN targets. To verify the circPTN sponging of miR-145-5p and miR-330-5p, we performed the following experiments: i. After inserting the sequences of wild type circPTN or miR-145-5p/miR-330-5p binding sites (Mut1/Mut2) into the 3’UTR of psiCHECK2 to construct a dual-luciferase reporter plasmid, we found that the luciferase activity was significantly decreased after co-transfection of miR-145-5p/miR-330-5p with the wild type plasmid, whereas no change in luciferase activity occurred with Mut1/Mut2. ii. By performing RNA-RNA pull-down assays by using biotin-labeled circPTN, we determined that miR-145-5p/miR-330-5p was markedly increased in U251 cells compared with the controls. Iii. circPTN and miR-145-5p/miR-330-5p were shown to be co-localized by FISH. iv. After treatment with actinomycin D, we observed that miR-145-5p and miR-330-5p degraded more rapidly when circPTN was overexpressed compared with EV. v. SOX9 and ITGA5 are known targets of miR-145-5p and miR-330-5p, respectively [36–38]. We demonstrated that circPTN could rescue the downregulation of SOX9 and ITGA5 induced by miR-145-5p and miR-330-5p. Considering these results, we concluded that circPTN can sponge miR-145-5p and miR-330-5p. In addition, we demonstrated that the inhibition of proliferation by miR-145-5p/miR-330-5p could be rescued by enforcing expression of circPTN. Therefore, we propose that circPTN promotes proliferation by sponging miR-145-5p/miR-330-5p.

Because circPTN was shown to sponge miR-145-5p, which has been reported to be a negative regulator of maintaining stemness and self-renewal [36, 38, 40], and because circPTN expression was more than 10-fold higher in G15 cells compared with S15 cells, we hypothesized that circPTN may be a positive regulator of self-renewal. We performed tumor sphere formation assays and, as expected, we determined that circPTN promoted self-renewal. Moreover, circPTN increased the levels of stemness markers, such as Nestin, CD133, SOX2, and SOX9. The positive regulation of self-renewal and the maintenance of stemness did not occur after transfection with circPTN-145-mut, which incorporated mutated binding sites of miR-145-5p. We then used U87-luc-EV or U87-luc-circPTN, generated by lentiviral transfection, to establish a nude mouse intracranial xenograft model. We observed that the tumor- bearing mice in the circPTN group displayed more rapid tumor growth and had shorter survival times compared with the mice in the EV group. In conclusion, circPTN promoted tumor growth in vitro and in vivo, primarily via sponging miR-145-5p/miR-330-5p.

It is noteworthy that several studies showed lower expression of circRNAs in cancer tissues than that in normal tissues. These studies have included breast cancer, colorectal cancer, gastric cancer, hepatocellular carcinoma, prostate adenocarcinoma, and glioma [28, 42, 43]. As high proliferative activity occurs in cancer tissues, it may be the case in general that the number of circRNAs is negatively associated with proliferation. However, whether the alteration in the general number of circRNAs is the reason or the result of proliferation is still unknown. Bachmayr et al. hypothesized that the reduction in circRNAs results from their distribution from mother cells to daughter cells during proliferation [43]. It is our hypothesis that the alteration in circRNA number may partially influence proliferation. Each circRNA likely has the following two effects: a general effect, which can inhibit proliferation, and a unique effect that is determined by the specific sequence of the circRNA and may affect activities such as miRNA sponging, interaction with RNA-binding proteins, and the translation of proteins. The final effect of each circRNA would therefore represent the integration of these general and unique effects. While further research will be required to validate this hypothesis, the phenomenon wherein the number of circRNAs is negatively associated with proliferation does exist. Instead of using individual circRNAs as biomarkers, we should examine the general number of circRNAs in tumor tissue as a reflection of their proliferative activity, from which to predict prognosis to some extent.

CircRNAs are more stable than linear RNAs and, thus they have potential use as biomarkers of diagnosis or follow-up in cancers. A previous study reported that cancer-associated circRNAs were detected and highly stable in plasma exosomes in xenograft-bearing nude mice [44]. Furthermore, one of the non-small cell lung cancer fusion gene, EML4-ALK, which activates ALK kinase and contributes to poor prognosis, derives from the circRNA-circ F-EA, which can be detected in the plasma of patients who harbored the EML4-ALK fusion gene [45]. These findings suggest promising prospects for the application of circRNA as liquid biopsy markers for the diagnosis of cancer and instructions for clinical treatment. Therefore, we will continue to investigate whether circPTN has the potential as a biomarker for the diagnosis or follow-up of patients with glioma.

## Conclusions

In conclusion, our study demonstrates that circPTN is upregulated in glioma tissues compared with normal brain tissue and contributes to the high proliferation activity of glioma cells by sponging miR-145-5p and miR-330-5p. Moreover, circPTN sponges miR-145-5p to promote the self-renewal of glioma stem cells, which is associated with tumorigenesis in glioma.

## Additional files

Additional file 1: **Table S1.** Sequences of si-circPTN, primers and probes for FISH. (DOCX 19 kb)

Additional file 2: **Figure S1.** Stable transfection system in U87-GSC and U251-GSC. **A**. Images of U87-GSC and U251-GSC in non-serum cultivation. **B**. Stable transfection system of circPTN and sh-circPTN in U87-GSC and U251-GSC, *n* = 3,*P < 0.05, *t-test*; **C**. Stable transfection system of miR-145-5p, miR-330-5p and circPTN-145-mut in U87-GSC and U251-GSC, *n* = 3, *p < 0.05, *t-test*. (TIF 730 kb)

Additional file 3: **Figure S2.** circPTN promotes self- renewal of U87-GSC and U251-GSC via sponging miR-145-5p. **A**. Upper: Images of tumor sphere formation assay in U87-GSC and U251-GSC at day 14 (200 cell/well); scale bar, 50 μm; Lower: Analyses of tumor sphere formation results, *n* = 10, *p < 0.05, *t- test*. **B**. Left: Result of western blot assay of stemness markers in U87-GSC and U251-GSC. (TIF 3267 kb)

Additional file 4: **Figure S3.** circPTN rescues the inhibition of self- renewal by miR-145-5p in U87-GSC and U251-GSC. **A**. Results of western blot assay showed that circPTN could rescue the inhibition of SOX9 expression by miR-145-5p in U87-GSC and U251-GSC. **B**. Upper: Images of tumor sphere formation assay (200 cells/well) at day 14; scale bar, 50 μm; Lower: Analyses of tumor sphere formation results indicated that circPTN could rescue the inhibition of self-renewal of U87-GSC and U251-GSC by miR-145-5p, *n* = 10, *p < 0.05, *t test*. **C**. Results of western blot assay showed that circPTN-145-mut failed to rescue the inhibition of SOX9 expression by miR-145-5p in U87-GSC and U251-GSC. **D**. Upper: Images of tumor sphere formation assay (200 cells/well) at day 14; scale bar, 50 μm; Lower: Analyses of tumor sphere formation results indicated that circPTN-145-mut failed to rescue the inhibition of self-renewal of U87-GSC and U251-GSC by miR-145-5p, *n* = 10, *p < 0.05, *t test*. (TIF 2388 kb)

## Data Availability

The datasets during and/or analyzed during the current study are available from the corresponding author on reasonable request.

## Abbreviations

circRNA: Circular RNA
GSCs: glioma stem cell
miRNA: microRNA
NBT: normal brain tissues
