# Supplementary material for: circPTN sponges miR-145-5p/miR-330-5p to promote proliferation and stemness in glioma
Source: J Exp Clin Cancer Res. 2019 Sep 11;38:398. doi: 10.1186/s13046-019-1376-8 (PMC6737709; doi:10.1186/s13046-019-1376-8)
Supplement: Supplementary file 1 — Table S1. Sequences of si-circPTN, primers and probes for FISH. (DOCX 19 kb) [file 13046_2019_1376_MOESM1_ESM.docx]

| Sequences of siRNAs | | | | | |
| --- | --- | --- | --- | --- | --- |
| siRNA-1 | | CTCAAGAATGCAGGCTCAA | | | |
| siRNA-2 | | TCAAGAATGCAGGCTCAAC | | | |
| siRNA-3 | | CCTCAAGAATGCAGGCTCA | | | |
| siRNA-4 | | CAAGAATGCAGGCTCAACA | | | |
| siRNA-5 | | CCAAACCTCAAGAATGCAG | | | |
| siRNA-6 | | CCCAAACCTCAAGAATGCA | | | |
| siRNA-7 | | AAACCTCAAGAATGCAGGC | | | |
| siRNA-8 | | AACCTCAAGAATGCAGGCT | | | |
| siRNA-9 | | CAAACCTCAAGAATGCAGG | | | |
| Sequences of primers for PCR | | | | | |
| circPTN-1 | | | Forward primer | AACTGGAAGTCTGAAGCGAGC | |
|  |  |  | Reward primer | TGTTGAGCCTGCATTCTTGAG | |
| circPTN-2  (Amplicate circPTN-145-mut) | | | Forward primer | CAAGCCCTGTGGCAAACTGAC | |
|  |  |  | Reward primer | TTCGACGCTGCTGCTGGTAC | |
| PTN | | | Forward primer | AACTGACCAAGCCCAAACCT | |
|  |  |  | Reward primer | GGTGACATCTTTTAATCCAGCA | |
| SOX9 | | | Forward primer | AGTACCCGCACTTGCACAAC | |
|  |  |  | Reward primer | CGTTCTTCACCGACTTCCTC | |
| SOX2 | | | Forward primer | GCTACAGCATGATGCAGGACCA | |
|  |  |  | Reward primer | TCTGCGAGCTGGTCATGGAGTT | |
| Nestin | | | Forward primer | AGCAGGAGGAGTTGGGTTCT | |
|  |  |  | Reward primer | AGGGGAGTGGAGTCTGGAAG | |
| CD133 | | | Forward primer | GCAATCTCCCTGTTGGTGA | |
|  |  |  | Reward primer | CCAGTTTCCGACTCCTTTTG | |
| GADPH | | | Forward primer | GCACCGTCAAGGCTGAGAAC | |
|  |  |  | Reward primer | TGGTGAAGACGCCAGTGGA | |
| circSMO | | | Forward primer | GCTACTTCCTCATCCGAGAATG | |
|  |  |  | Reward primer | ACCAAGGGCACTTCGCACT | |
| circCLIP2 | | | Forward primer | GCACAGCATGAGCAGGTACTTC | |
|  |  |  | Reward primer | CGATACGGATCACTTTGTGGATG | |
| circPLOD2 | | | Forward primer | GATGATCTGGTTGTCATGTTTACTG | |
|  |  |  | Reward primer | CCATCACTTTCTTTTGTTGCTACAG | |
| circTEX9 | | | Forward primer | CAACAGTTGTCTTCAGTAGAAAGGAG | |
|  |  |  | Reward primer | GGTCTTTGGCTTAGTTTCTGAATGA | |
| circZBTB20 | | | Forward primer | CACATGTTCGTACACACAGGTGA | |
|  |  |  | Reward primer | GGAATTGCTGAAGTTGTGAAGGTT | |
| circVCAN | | | Forward primer | TGAAGAGTCAGTGGAAGGCAC | |
|  |  |  | Reward primer | CAGCCATTAGATCATGCACTGGA | |
| circSOX6 | | | Forward primer | TGGGACAGCGTTCTGTCATC | |
|  |  |  | Reward primer | AGACATTCTTCCTCTTGTTCAGT | |
| circLPHN3  (hsa_circ_0069865) | | | Forward primer | CGAACTCCTGGACGCTACTC | |
|  |  |  | Reward primer | TGTCTCGACCATTGCCTCTG | |
| circFANCL | | | Forward primer | GCTGTATGCACTACCTCCTCC | |
|  |  |  | Reward primer | GTGGAAGTCTCTTCCTTATCCCA | |
| circLPHN3  (hsa_circ_0126761) | | | Forward primer | TGATAACATCAAGCAATGGTCG | |
|  |  |  | Reward primer | CGTAGTCAGGTCTCTCCATGC | |
| circKDM4B | | | Forward primer | CAATCACGGGTTCAACTGCG | |
|  |  |  | Reward primer | CACTGGGCCACGTCCTG | |
| circMKLN1 | | | Forward primer | ACAAGCTGGTGTTGAAGGGT | |
|  |  |  | Reward primer | AGCAGGCCTTTCGAGCTTTA | |
| circWDR78 | | | Forward primer | TAGCAGAAGACCTGGAAGAACC | |
|  |  |  | Reward primer | TGTGGCATTGTTCCTGTGAA | |
| Probes for FISH | | | | | |
|  | probe | | | | label |
| circPTN | GCCAGTATGAAAATGAATGCCAAGAAGGCAGCTGCAAATTTTCGACGCTGCTGCTGGTACTGTTGAGCCTGCATTCTTGAGGTTTGGGCTTGGTCAGTTTGCCACAGGGCTTGGAGATGGTGACAGTCTTCTGGCATTCGGCATTGTGCAGGGC | | | | Cy3 |
| miR-145-5p | AGGGAUUCCUGGGAAAACUGGAC | | | | FAM |
| miR-330-5p | GCCUAAGACACAGGCCCAGAGA | | | | FAM |
