# Supplementary figures and images for: circPTN sponges miR-145-5p/miR-330-5p to promote proliferation and stemness in glioma
Source: J Exp Clin Cancer Res. 2019 Sep 11;38:398. doi: 10.1186/s13046-019-1376-8 (PMC6737709; doi:10.1186/s13046-019-1376-8)

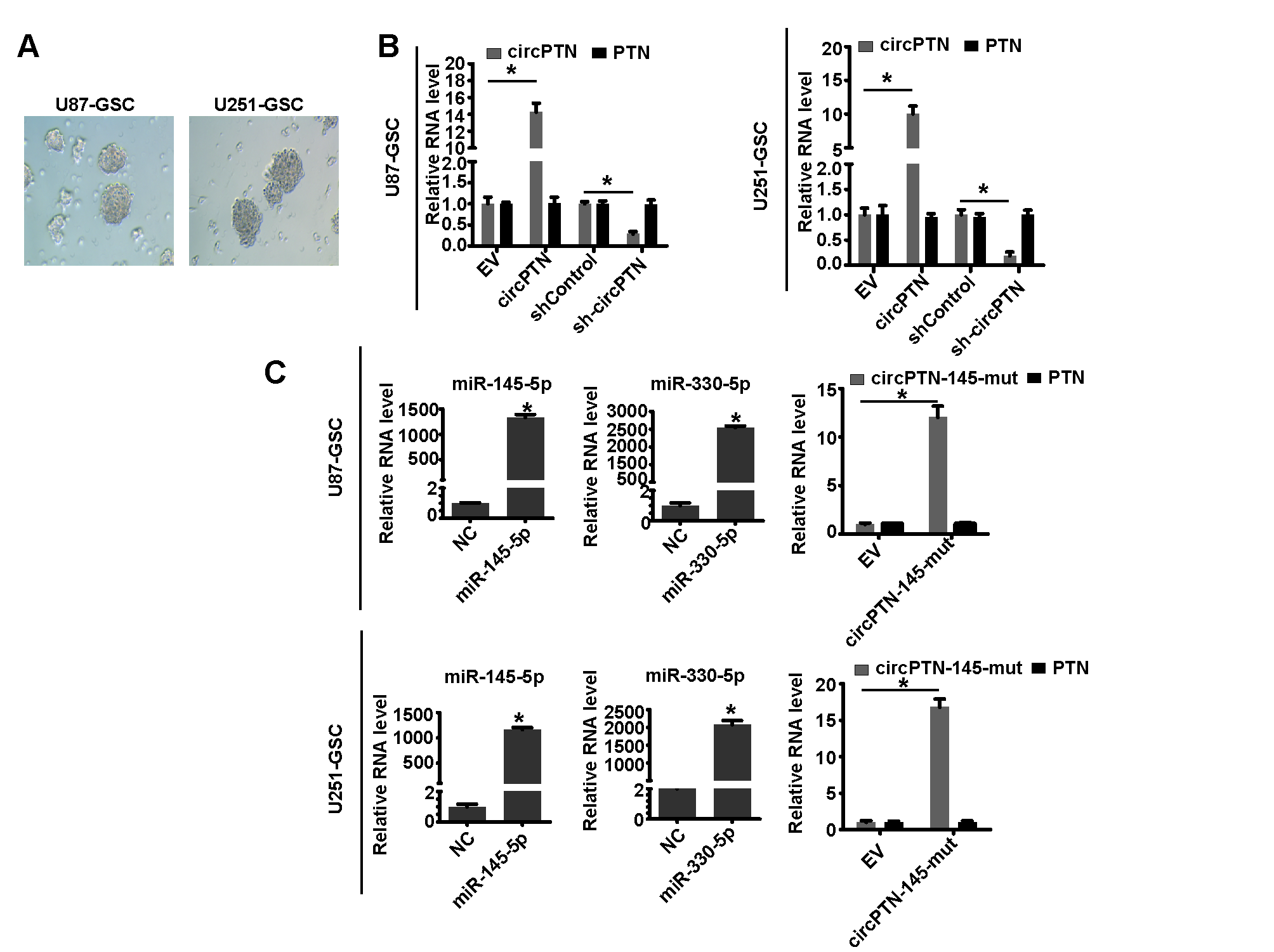

Supplement: Supplementary file 2 — Figure S1. Stable transfection system in U87-GSC and U251-GSC. A. Images of U87-GSC and U251-GSC in non-serum cultivation. B. Stable transfection system of circPTN and sh-circPTN in U87-GSC and U251-GSC, n = 3,*P < 0.05, t-test; C. Stable transfection system of miR-145-5p, miR-330-5p and circPTN-145-mut in U87-GSC and U251-GSC, n = 3, *p < 0.05, t-test. (TIF 730 kb) [file 13046_2019_1376_MOESM2_ESM.tif]

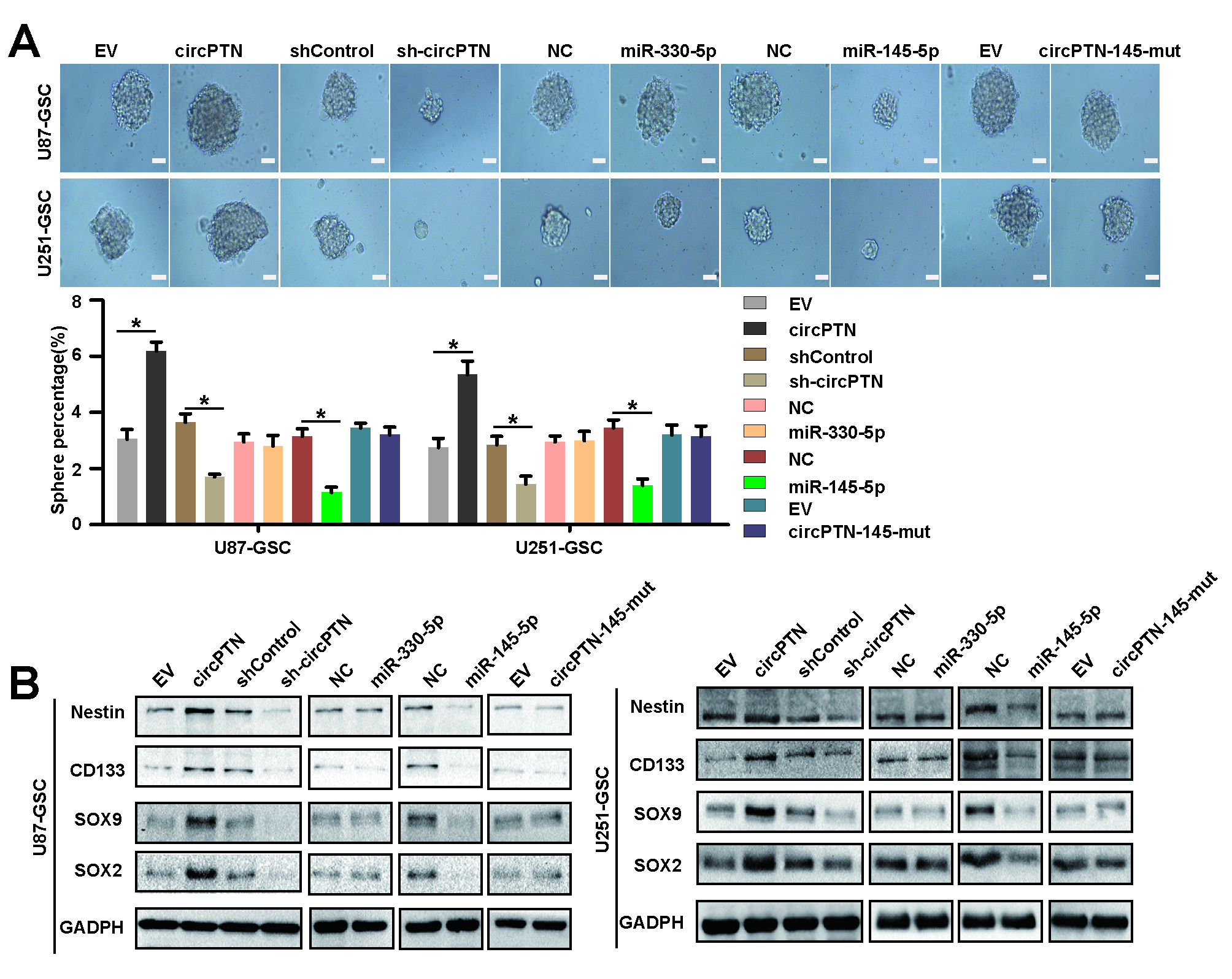

Supplement: Supplementary file 3 — Figure S2. circPTN promotes self- renewal of U87-GSC and U251-GSC via sponging miR-145-5p. A. Upper: Images of tumor sphere formation assay in U87-GSC and U251-GSC at day 14 (200 cell/well); scale bar, 50 μm; Lower: Analyses of tumor sphere formation results, n = 10, *p < 0.05, t- test. B. Left: Result of western blot assay of stemness markers in U87-GSC and U251-GSC. (TIF 3267 kb) [file 13046_2019_1376_MOESM3_ESM.tif]

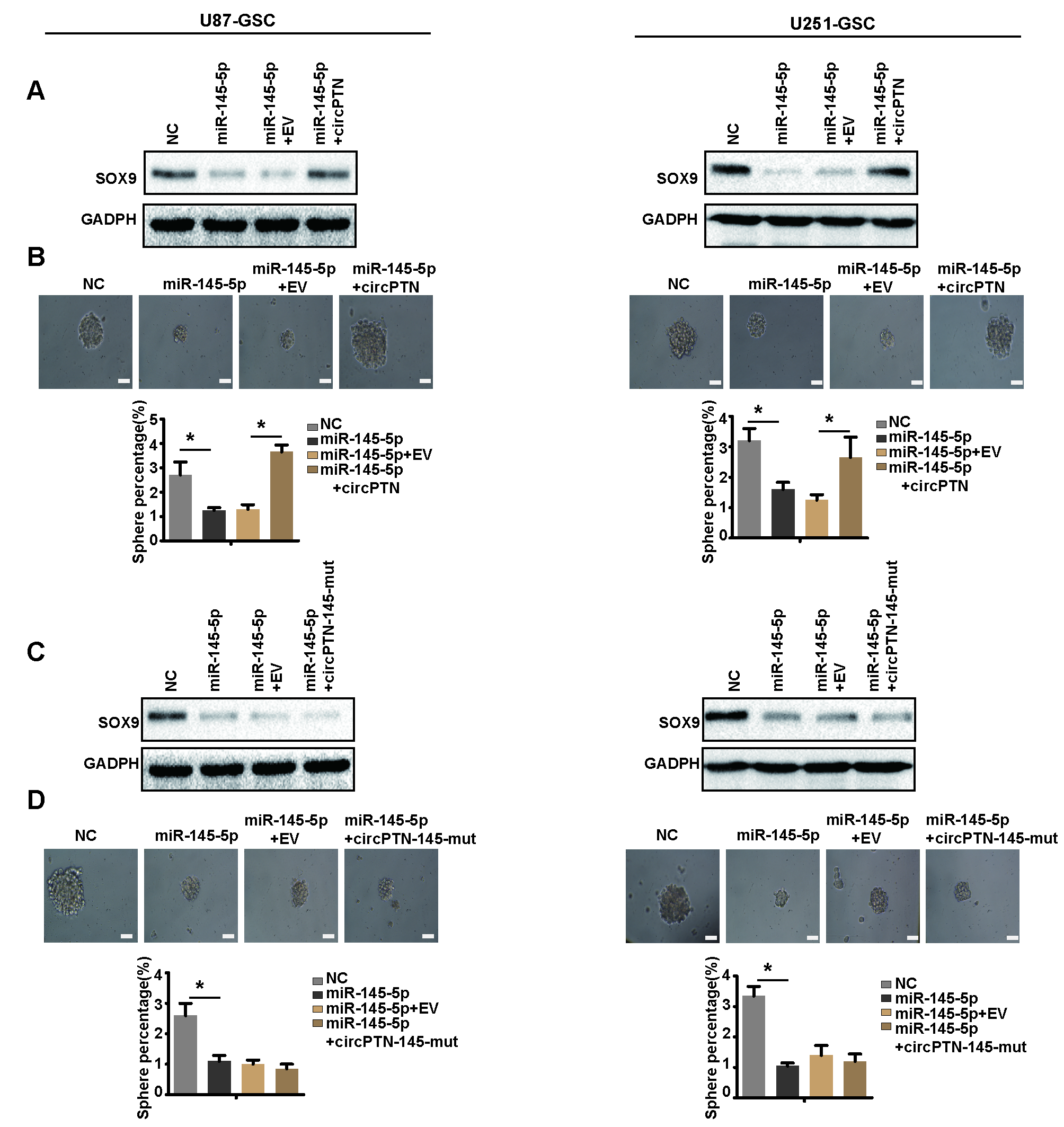

Supplement: Supplementary file 4 — Figure S3. circPTN rescues the inhibition of self- renewal by miR-145-5p in U87-GSC and U251-GSC. A. Results of western blot assay showed that circPTN could rescue the inhibition of SOX9 expression by miR-145-5p in U87-GSC and U251-GSC. B. Upper: Images of tumor sphere formation assay (200 cells/well) at day 14; scale bar, 50 μm; Lower: Analyses of tumor sphere formation results indicated that circPTN could rescue the inhibition of self-renewal of U87-GSC and U251-GSC by miR-145-5p, n = 10, *p < 0.05, t test. C. Results of western blot assay showed that circPTN-145-mut failed to rescue the inhibition of SOX9 expression by miR-145-5p in U87-GSC and U251-GSC. D. Upper: Images of tumor sphere formation assay (200 cells/well) at day 14; scale bar, 50 μm; Lower: Analyses of tumor sphere formation results indicated that circPTN-145-mut failed to rescue the inhibition of self-renewal of U87-GSC and U251-GSC by miR-145-5p, n = 10, *p < 0.05, t test. (TIF 2388 kb) [file 13046_2019_1376_MOESM4_ESM.tif]
